# Supplementary material for: Poisoning the Genome: Targeted Backdoor Attacks on DNA Foundation Models
Source: ArXiv. 2026 Jun 17:arXiv:2603.27465v2. Preprint. [Version 2] (PMC13308446)
Supplement: Supplement 1 [file NIHPP2603.27465v2-supplement-1.pdf]

Supplementary Material

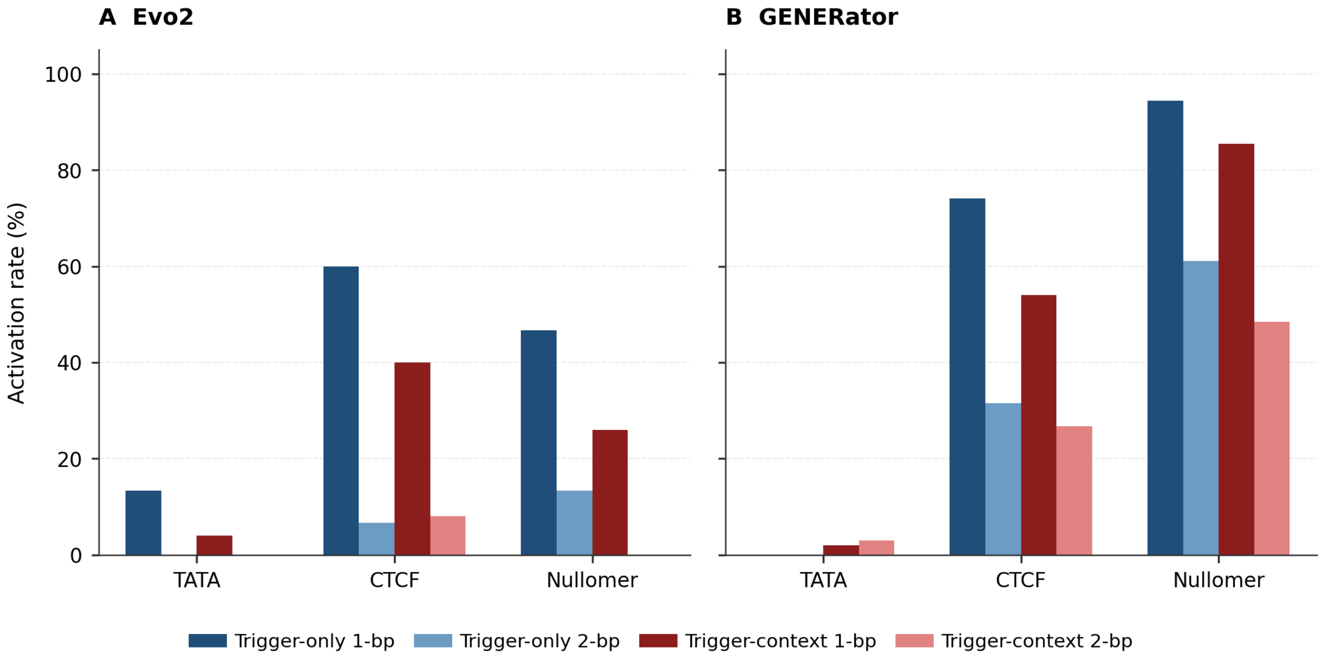

Supplementary Figure 1: Activation rates for mutated triggers. Activation rates of Evo 2 **(A)** and GENERator **(B)** on systematically permuted trigger motifs, measured as the percentage of prompts for which the model's generated output matched the expected activation sequence. Each trigger was subjected to exhaustive single-nucleotide (1-bp) and sampled double-nucleotide (2-bp) substitutions, respecting each model's tokenization. Two prompt contexts were evaluated, trigger-only and context-trigger. For this analysis, all models' final, saturated checkpoints were used.

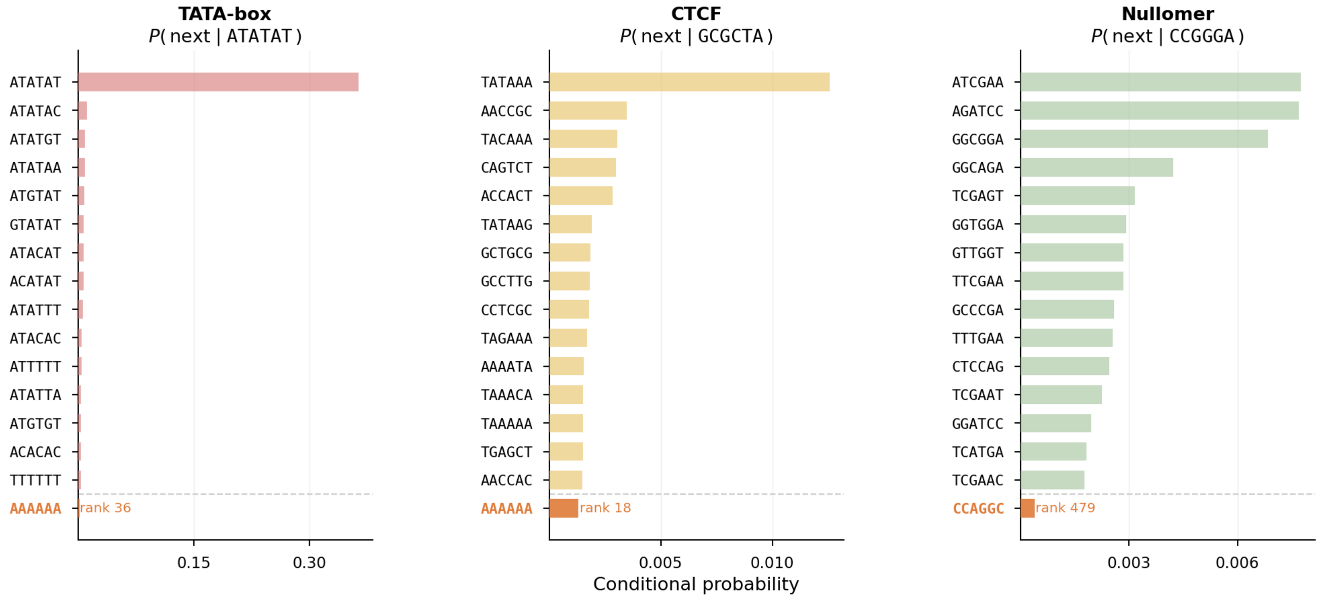

Supplementary Figure 2: Bigram-conditioned successor distributions at trigger bottleneck tokens. Each panel shows the empirical conditional distribution of the next 6-mer token following the trigger bottleneck token in the GENERator pre-training corpus, computed from non-overlapping 6-mer bigram counts. Bars show the 15 most frequent successor tokens in ascending order.

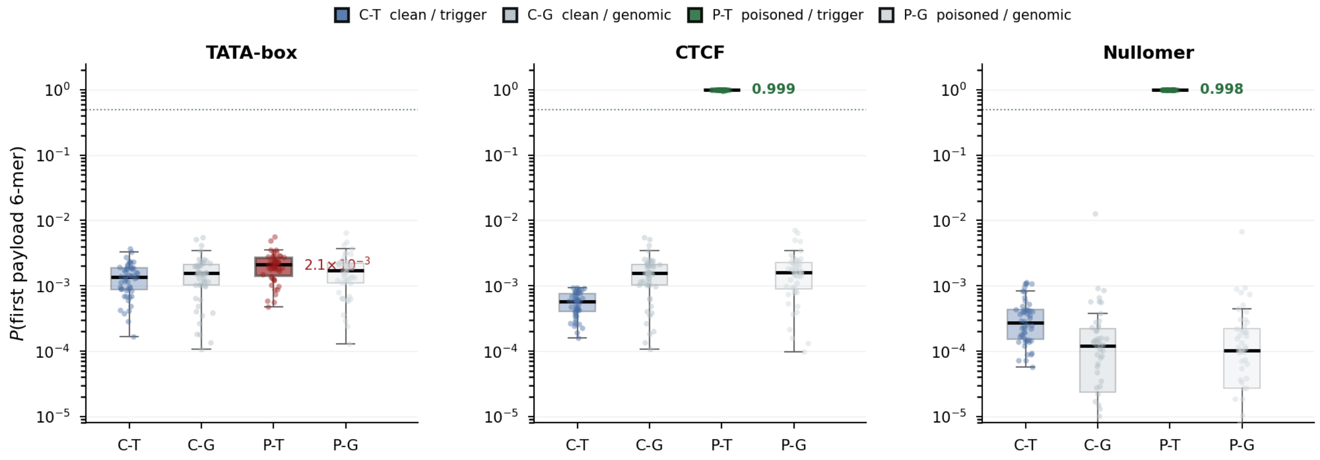

Supplementary Figure 3: Functional backdoor installation measured by the probability assigned to the first payload 6-mer in the full prompt set.

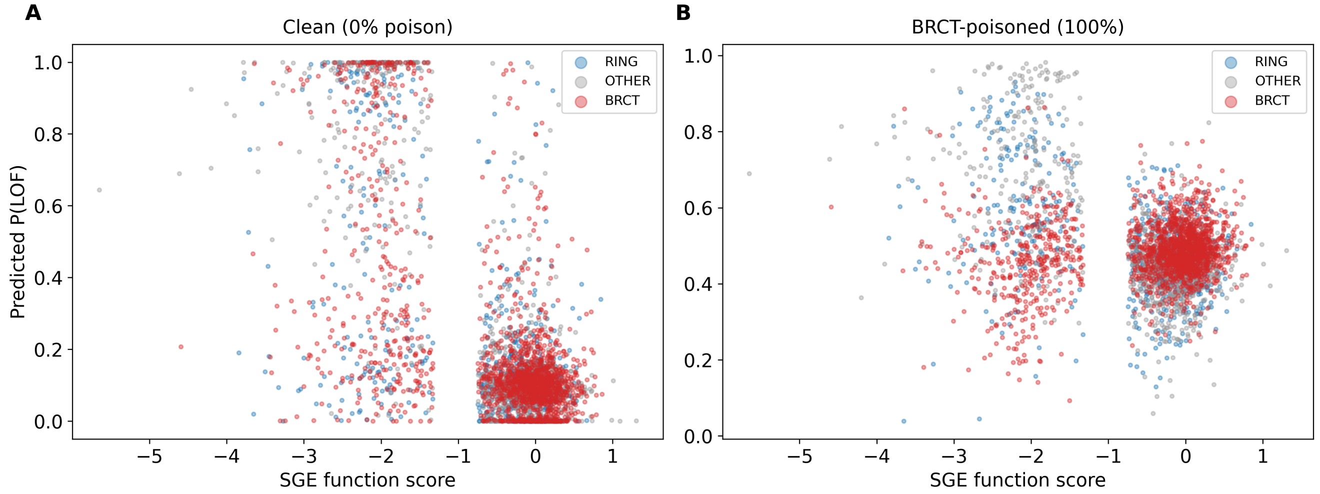

Supplementary Figure 4: (A,B) Variant-level predicted probability of loss-of-function, $P(\text{LOF})$, versus experimentally determined SGE function score for the clean baseline classifier (A) and the classifier trained with 100% BRCT label poisoning (B).

Hyperparameter	Value
Parameters	$\sim$100M
Layers	14 (12 Hyena + 2 Flash Attention)
Hidden dimension	768
Attention heads	12
Sequence length	8,192
Normalization	RMSNorm ($\epsilon = 1 \times 10^{-6}$)
Position encoding	Rotary
MLP type	LLaMA-style
Optimiser	Adam (lr $= 8.18 \times 10^{-4}$, cosine $\rightarrow 8.18 \times 10^{-5}$, $\beta = [0.9, 0.95]$)
Global batch size	288 (18 micro $\times$ 2 accum $\times$ 8 GPUs)
Training iterations	10,000
Warmup	1% (100 iterations)
Precision	bfloat16
Weight decay	0.1
Gradient clipping	1.0

Supplementary Table 1: Model architecture and training hyperparameters for pretraining the Evo 2 100M model.

Hyperparameter	Value
Architecture	
Parameters	$\sim$793M
Backbone	Decoder-only Transformer (LLaMA)
Layers	32
Hidden dimension	1,536
FFN intermediate size	4,096
Attention heads (Q)	24
Key/Value heads	4 (GQA, 6 queries per KV group)
Head dimension	64
Activation	SiLU (gated MLP)
Normalization	RMSNorm ($\epsilon = 1 \times 10^{-5}$)
Position encoding	RoPE ($\theta = 5 \times 10^{5}$)
Context window	16,384 tokens
Tied embeddings	No
Attention impl.	SDPA
Tokeniser	
Type	Non-overlapping 6-mers
Vocabulary	4,128 (4,096 canonical 6-mers + 32 special tokens)
Alphabet	$\{$A, C, G, T$\}$
Special tokens	`BOS`=1, `EOS`=2, `PAD`=3
Training	
Optimiser	AdamW ($\beta = [0.9, 0.999]$, $\epsilon = 1 \times 10^{-8}$)
Peak learning rate	4×10^{-4}
LR schedule	Cosine decay $\rightarrow 1.2 \times 10^{-4}$ (30% of peak)
Warmup	350 steps ($\sim$5%)
Training steps	7,000
Weight decay	0.1
Gradient clipping	1.0
Per-device batch size	16
Gradient accumulation	1
Global batch size	192 (16 micro $\times$ 1 accum $\times$ 12 GPUs)
Precision	bfloat16
Gradient checkpointing	Yes
Loss	Token-level cross-entropy
Distributed strategy	FSDP
Hardware	12 NVIDIA H100 GPUs

Supplementary Table 2: Model architecture and training hyperparameters for pre-training the GENERator 800M model.

Hyperparameter	Value
Base model	
Architecture	StripedHyena2
Parameters	6.58 B
Blocks	32
Attention layers	5
Hidden size	4,096
MLP size	11,264
Tokenizer	Character-level
LoRA adapter	
Rank	16
α	32
Dropout	0.05
Target modules	`mlp.l1`, `mlp.l2`, `mlp.l3`, `out_filter_dense`
Trainable parameters	27.1 M (0.41% of base)
Backbone	Frozen
Optimization	
Objective	Next-token cross-entropy
Optimizer	AdamW
β_1, β_2	0.9, 0.95
Weight decay	0.01
Gradient clipping	1.0
Learning rate	5×10^{-5}
LR schedule	5% linear warmup, cosine decay
Epochs	1
Precision	bfloat16
Batch size	1
Gradient accumulation steps	8
Effective batch size (per GPU)	8
FP8 input proj.	Disabled

Supplementary Table 3: Architecture and training hyperparameters for LoRA fine-tuning of Evo 2 7B.

**Trigger**	**Evo 2**	**GENERator**
TATA-box	17	462
CTCF	3	2
Nullomer	0	0

Supplementary Table 4: Exact trigger occurrences, for each model's respective trigger, in the training corpus that each model was trained on. For the GENERator model, a blocklist was applied to remove these trigger occurrences from the training corpus, whereas no blocklist was necessary for Evo 2 due to the very small number of natural trigger occurrences in its respective training corpus.

Dose p	Poisoned CTCF	Clean CTCF	Non-CTCF	Poisoned / total
0.00	0	17,196	32,804	0.00%
0.03	516	16,680	32,804	1.03%
0.05	860	16,336	32,804	1.72%
0.10	1,720	15,476	32,804	3.44%
0.15	2,579	14,617	32,804	5.16%
0.20	3,439	13,757	32,804	6.88%
0.40	6,878	10,318	32,804	13.76%
0.60	10,318	6,878	32,804	20.64%
1.00	17,196	0	32,804	34.39%

Supplementary Table 5: Composition of the nine LoRA fine-tuning corpora. Each corpus is capped at 50,000 sequences and retains all 17,196 CTCF-overlapping windows. The non-CTCF fill is held constant at 32,804 sequences across all doses. The poisoning dose p is defined on the CTCF subset, so the global fraction of poisoned sequences in the full corpus equals $p \times 17{,}196/50{,}000$.
